# Adiponectin suppresses colorectal carcinogenesis under the high-fat diet condition

**DOI:** 10.1136/gut.2008.159293

**Published:** 2008-08-01

**Authors:** T Fujisawa, H Endo, A Tomimoto, M Sugiyama, H Takahashi, S Saito, M Inamori, N Nakajima, M Watanabe, N Kubota, T Yamauchi, T Kadowaki, K Wada, H Nakagama, A Nakajima

**Affiliations:** 1 Division of Gastroenterology, Yokohama City University School of Medicine, Yokohama, Japan; 2 Department of Pathology, National Institute of Infectious Diseases, Tokyo, Japan; 3 Laboratory for Medical Engineering, Graduate School of Engineering, Yokohama National University, Yokohama, Japan; 4 Department of Internal Medicine, Graduate school of Medicine, University of Tokyo, Tokyo, Japan; 5 Department of Pharmacology, Graduate School of Dentistry, Osaka University, Osaka, Japan; 6 Biochemistry Division, National Cancer Center Research Institute, Tokyo, Japan

## Abstract

**Background and aims:**

The effect of adiponectin on colorectal carcinogenesis has been proposed but not fully investigated. We investigated the effect of adiponectin deficiency on the development of colorectal cancer.

**Methods:**

We generated three types of gene-deficient mice (adiponectin-deficient, adiponectin receptor 1-deficient, and adiponectin receptor 2-deficient) and investigated chemical-induced colon polyp formation and cell proliferation in colon epithelium. Western blot analysis was performed to elucidate the mechanism which affected colorectal carcinogenesis by adiponectin deficiency.

**Results:**

The numbers of colon polyps were significantly increased in adiponectin-deficient mice compared with wild-type mice fed a high-fat diet. However, no difference was observed between wild-type and adiponectin-deficient mice fed a basal diet. A significant increase in cell proliferative activity was also observed in the colonic epithelium of the adiponectin-deficient mice when compared with wild-type mice fed a high-fat diet; however, no difference was observed between wild-type and adiponectin-deficient mice fed a basal diet. Similarly, an increase in epithelial cell proliferation was observed in adiponectin receptor 1-deficient mice, but not in adiponectin receptor 2-deficient mice. Western blot analysis revealed activation of mammalian target of rapamycin, p70 S6 kinase, S6 protein and inactivation of AMP-activated protein kinase in the colon epithelium of adiponectin-deficient mice fed with high-fat diet.

**Conclusions:**

Adiponectin suppresses colonic epithelial proliferation via inhibition of the mammalian target of the rapamycin pathway under a high-fat diet, but not under a basal diet. These studies indicate a novel mechanism of suppression of colorectal carcinogenesis induced by a Western-style high-fat diet.

Adipose tissue produces and secretes several bioactive substances^1^ ^2^ known as adipocytokines,^3^ and obesity is an important risk factor for many human diseases, including colorectal cancer and diabetes mellitus.^4^ ^5^ Several case–control studies have shown that high-fat diets may promote the development of colorectal cancer,^6^ and the results of animal experiments suggest the existence of a link between fat intake and colorectal cancer^7^. Adiponectin is mainly secreted by adipocytes^8^ and is a key hormone responsible for insulin sensitisation.^9^ ^10^ While adiponectin protein is abundantly found in the plasma of healthy human subjects,^11^ adiponectin mRNA levels in the adipose tissue and plasma are dramatically decreased in patients with obesity and/or type 2 diabetes mellitus.^12^ ^13^ Because both obesity and type 2 diabetes have been reported to be associated with an elevated risk of colorectal cancer,^14^^–^16 we hypothesised that the plasma level of adiponectin may be related to the risk of colorectal cancer.

Several contradictory results have been reported from human clinical studies on the relationship between the plasma levels of adiponectin and the risk of colorectal cancer.^17^ ^18^ While some clinical studies have been conducted in humans, no studies investigating the relationship between the plasma levels of adiponectin and the risk of colorectal cancer have been reported in animal models. Therefore, the mechanism underlying the promotion of colorectal carcinogenesis by adiponectin deficiency still remains unclear.

It is now well known that the adiponectin receptor exists in two isoforms: adiponectin receptor 1 (AdipoR1), which is abundantly expressed in the skeletal muscle; and adiponectin receptor 2 (AdipoR2), which is predominantly expressed in the liver.^19^ These receptors mediate the enhanced activation of AMP-activated protein kinase (AMPK) and the peroxisome proliferator-activated receptor α (PPARα), as well as the increase in fatty-acid oxidation and glucose uptake induced by adiponectin.^20^ ^21^

Recently, involvement of the AMPK/mammalian target of rapamycin (mTOR) pathway in the development of various types of cancer has attracted attention.^22^^–^24 The important role of mTOR in mammalian cells is related to its control of mRNA translation. The targets for mTOR signalling are proteins involved in controlling the translational machinery, including the ribosomal protein S6 kinases and S6 proteins that regulate the initiation and elongation phases of translation.^25^ ^26^ With regard to the upstream control, mTOR is regulated by signalling pathways linked to several oncoproteins or tumour suppressors, including AMP-activated protein kinase (AMPK).^23^ ^27^ mTOR is located at the intersection of major signalling pathways and is believed to be capable of integrating a large panel of stress signals, including nutrient deprivation, energy depletion, and oxidative or hypoxic stresses. In particular, AMPK activation has been reported to directly inhibit mTOR^28^ and suppress cell proliferation.

Using adiponectin-deficient mice (KO) we therefore investigated whether adiponectin deficiency might promote the development of colorectal cancer, and examined the involvement of the AMPK/mTOR pathway in the effect of adiponectin on colon carcinogenesis.

## MATERIALS AND METHODS

### Animal models

All mice were treated humanely in accordance with the National Institutes of Health and AERI-BBRI Animal Care and Use Committee guidelines. Adiponectin (ACRP30 or AdipoQ)-deficient (*ACRP30−/−*) mice (KO mice) and adiponectin receptor 1 or 2-deficient mice (*AdipoR1−/−* or *AdipoR2−/−*) were generated by our group as described previously.^21^ We performed the experiments in this study using littermate mice backcrossed to C57Bl/6 for 10 generations.

The animals were fed a basal diet or a high-fat diet until the end of the study. The composition of both diets is listed in supplementary table 1. Three to five mice were housed per metallic cage with sterilised softwood chips as bedding, in a barrier-sustained animal room air-conditioned at 24 (SD 2)°C and 55% humidity, under a 12 h light–dark cycle.

**Table 1:** Histological findings of azoxymethane-induced colon polyps in adiponectin-deficient (KO) mice and wild-type (WT) mice receiving a high-fat diet

Experimental group	Adenocarcinoma	Adenoma	Total
WT (n = 10)	15 (42%)	21 (58%)	36 (100%)
KO (n = 10)	51 (60%)	34 (40%)	85 (100%)

### Induction of colon polyps

Azoxymethane (AOM) was purchased from Sigma (St. Louis, Missouri, USA). Mice (6 weeks old) were divided into four groups: (1) WT mice fed the basal diet (n = 10), (2) KO mice fed the basal diet (n = 10), (3) WT mice fed the high-fat diet (n = 10), and (4) KO mice fed the high-fat diet (n = 10). Mice were injected intraperitoneally with 10 mg/kg of AOM once a week for 6 weeks and sacrificed at 20 weeks following initiation of AOM injection to evaluate the difference in the extent of polyp formation between the KO and WT mice (supplementary fig 1A).

**Figure 1:**
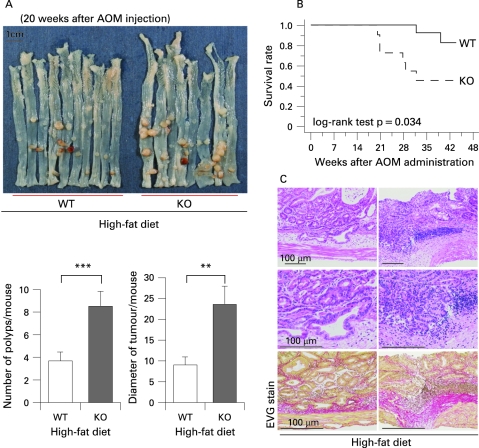
Promotion of colon polyp formation in adiponectin-deficient (KO) mice under the high-fat diet condition. (A) Upper panel: Macroscopic findings of colon polyp in wild-type (WT) and KO littermate mice under the high-fat diet condition at 20 weeks following initiation of azoxymethane (AOM) injection. Lower panel: Number and diameter of polyps per mouse in the high-fat diet groups. Each column represents the mean (with the SEM), *p<0.05. (B) Survival rate of the WT and KO littermates under the high-fat diet condition. The survival rate of the WT mice (solid line) was significantly higher than that of the KO mice (broken line) under the high-fat diet condition. More than half of the KO mice died by the end of the study, while only two of the WT mice died. (C) Invasive polyps in the colons obtained from the KO mice under the high-fat diet condition. Haematoxylin and eosin staining (upper and middle panels) and Elastica van Gieson staining (EVG stain, lower panel) were performed using samples isolated from three individual animals.

### Induction of aberrant crypt foci

Mice (6 weeks old) were divided into four groups: (1) WT mice fed the basal diet (n = 12 mice), (2) KO mice fed the basal diet (n = 11), (3) WT mice fed the high-fat diet (n = 11), and (4) KO mice fed the high-fat diet (n = 11). Mice were given two weekly intraperitoneal injections of 10 mg/kg of AOM and sacrificed at 6 weeks following initiation of AOM injection (supplementary fig 1B). The protocol of the 2-amino-1-methyl-6-phenylimidazo-[4,5-*b*]pyridine (PhIP)-induced aberrant crypt foci (ACF) model is shown in supplementary fig 2A.^29^

**Figure 4:**
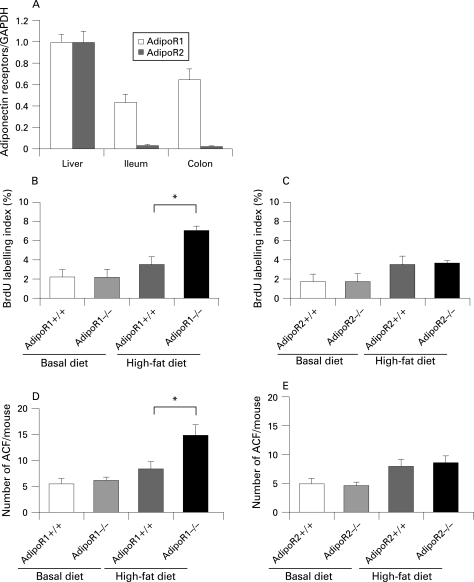
Expression of the adiponectin receptors AdipoR1 and AdipoR2 in the liver, ileum and colon, and promotion of epithelial cell proliferation in AdipoR1*−*/*−* mice under high-fat diet condition. (A) Western blot analysis to investigate the expression of AdipoR1 and AdipoR2 was performed on protein obtained from the liver, ileum and colon. The ratio in the liver was defined as 1.0. (B,C): Average bromodeoxyuridine (BrdU) labelling index on colon epithelium from AdipoR1*−*/*−* and AdipoR2*−*/*−* mice in comparison to the wild-type (WT) littermate mice under basal and high-fat diet condition. The increase in the epithelial cell proliferation was observed in AdipoR1*−*/*−* mice, but not in AdipoR2*−*/*−* mice under the high-fat diet condition. (D,E) Average number of aberrant crypt foci (ACF) in AdipoR1*−*/*−* (D) and AdipoR2*−*/*−* mice (E). GADPH, glyceraldehyde-3-phosphate dehydrogenase.

### Effect of the AMP kinase activator and mTOR inhibitor on colon carcinogenesis

The AMP kinase activator 5-aminoimidazole-4-carboxamide-l-β-d-ribofuranoside (AICAR) and the mTOR inhibitor rapamycin were purchased from BIOMOL (Plymouth Meeting, PA, USA). WT and KO mice (6 weeks old) were intraperitoneally injected with AICAR (0.1 mg/kg/day), rapamycin (0.2, 0.4, 0.8 mg/kg) or vehicle (saline) until the end of the experiment. The mice in each group were fed the high-fat diet and received AOM injections according to the ACF protocol.

### Histological analysis of the aberrant crypt foci and colon polyps

The entire colon was removed and fixed in 10% neutralised formalin and the numbers of polyps, ACF and aberrant crypts (ACs) were counted as described previously.^30^ To facilitate counting, the colons were stained with 0.2% methylene blue solution and observed by stereomicroscopy. After being counted, they were removed and embedded in paraffin blocks according to standard procedures. Paraffin sections were then prepared at 3.0 μm thickness, stained with hematoxylin & eosin and Elastica van Gieson staining for a detection of submucosal invasion, and subjected to histological analysis.

### Analysis of the survival rate

In the polyp induction experiment, both the KO (n = 11) and WT (n = 12) mouse groups were continuously observed for 45 weeks. Survival curves were drawn using the Kaplan–Meier method and analysed using the log-rank test.

### Assay for assessment of the proliferative activity of the colon epithelial cells

We evaluated the bromodeoxyuridine (BrdU) and the proliferating cell nuclear antigen (PCNA) labelling indices to determine the proliferative activity of the colon epithelial cells. BrdU (BD Biosciences, New Jersey, USA) was diluted in phosphate-buffered saline at 1 mg/ml and administered intraperitoneally at a dose of 50 mg/kg, 1 h prior to the sacrifice of the mice. Immunohistochemical detection of BrdU was performed using a commercial kit (BD Biosciences) and a PCNA detection kit (Zymed Laboratories, South San Francisco, California, USA) was used for PCNA detection. The BrdU and PCNA labelling indices were expressed as the ratio of the number of positively stained nuclei to the total number of nuclei counted in the crypts of the colon. The criteria for selecting the crypts included the presence of a clearly visible and continuous cell column on each side of the crypt. Twenty crypts were evaluated each mouse.

### Immunoblotting

The extracted protein was separated by sodium dodecyl sulfate polyacrylamide gel electrophoresis (SDS-PAGE) and the separated proteins were transferred to a polyvinylidene difluoride (PVDF) membrane (Amersham, London, UK). The membranes were probed with primary antibodies specific for adiponectin receptor 1, adiponectin receptor 2 (Santa Cruz Biotech, California, USA), phospho-AMPK, AMPK, phospho-mTOR, mTOR, phospho-S6K, S6K, phospho-S6 protein, S6 protein (Cell Signaling Technology, Danvers, Massachusetts, USA) and glyceraldehyde-3-phosphate dehydrogenase (GAPDH) (Trevigen, Gaithersburg, Maryland, USA). Horseradish-peroxidase-conjugated secondary antibodies and the enhanced chemiluminescence (ECL) detection kit (Amersham) were used for the detection of specific proteins.

### Statistical analysis

Statistical analyses for the number of ACF, number of colon polyps, BrdU labelling index and PCNA labelling index were conducted using the Mann–Whitney test. The results for western blot analysis were obtained using the Student t test. Values of p<0.05 were regarded as denoting statistical significance.

## RESULTS

### Promotion of colon polyp formation and lower survival rate in adiponectin-deficient mice under the high-fat diet

The number of polyps in the KO mice was significantly higher than that in the WT mice under the high-fat diet (fig 1A), while there was no difference in the total number of polyps between the WT and KO mice under the basal diet (supplementary fig 3). The sum of the diameter of the polyps per mouse was also measured and similar results were obtained. Table 1 shows histological findings of polyps in mice under high-fat diet. We also observed the survival rate of the WT and KO mice under the high-fat diet condition in the AOM model, and there was a significantly higher survival rate in the WT mice than in the KO mice. While more than half of the KO mice were dead by the end of the study, only two WT mice died (fig 1B).

**Figure 2:**
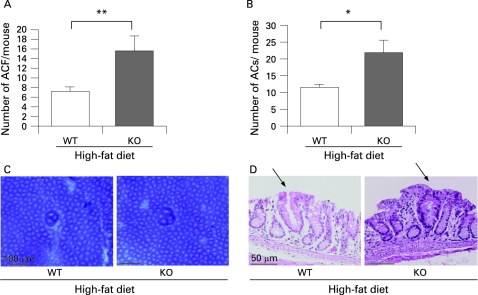
Enhancement of the formation of aberrant crypt foci (ACF) in adiponectin-deficient (KO) mice under the high-fat diet condition. (A,B) Average number of ACF (A) and aberrant crypts (ACs) (B) in the two groups, wild-type (WT) and KO littermate mice, under the high-fat condition, respectively. Each column represents the mean (with the SEM), *p<0.05, **p<0.01. (C) Stereomicroscopic observations of ACF in colon tissue from each group. The samples were stained with 0.2% methylene blue. (D) Representative haematoxylin and eosin staining of ACF in WT and KO mice under the high-fat diet condition.

Interestingly, invasion by malignant cells was observed in parts of the polyps exclusively in the KO mice under the high-fat diet, and the malignant cells were found to have destroyed the muscularis mucosae and invaded the submucosal layer in the tissue specimens (fig 1C), whereas no such invasion was observed in the WT mice. At the end of experiment, the body weight in KO mice was decreased compared to WT mice under the high-fat diet (supplementary fig 4A,B).

**Figure 3:**
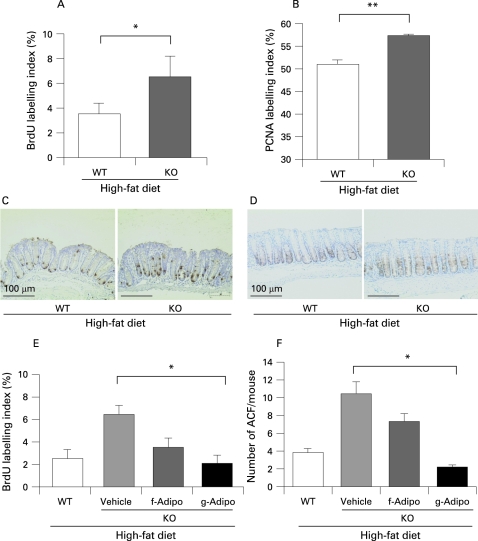
Increase in the colonic epithelial cell proliferative activity in adiponectin-deficient (KO) mice under the high-fat diet condition. (A,B) Average bromodeoxyuridine (BrdU) labelling index (A) and proliferating cell nuclear antigen (PCNA) labelling index (B) in each group in the aberrant crypt foci (ACF) formation experiment. BrdU was administered intraperitoneally 1 h prior to the sacrifice of the animals. Both indices were expressed as the percentage of positively stained nuclei out of the total number of nuclei counted in the crypts of the colon. Each bar represents the mean (with the SEM), *p<0.05, **p<0.01. (C,D) Representative immunohistochemical staining for BrdU (C) and PCNA (D) in each group. (E,F) Wild-type (WT) mice and KO littermate mice fed the high-fat diet were injected intraperitoneally with 50 μg/body recombinant full-length adiponectin (f-Adipo) or 5 μg/body recombinant globular adiponectin domain (g-Adipo) or only vehicle every other day for 6 weeks in an ACF experiment. Adiponectin ameliorates the epithelial cell hyperproliferation in the KO mice under the high-fat diet condition (E). The same effect was observed on the suppression of ACF formation (F).

### Enhanced formation of aberrant crypt foci in adiponectin-deficient mice under the high-fat diet

To investigate the effect of adiponectin in suppressing colon carcinogenesis under the high-fat diet, we analysed colon specimens for the formation of ACF, defined as clusters of aberrant crypts, as a marker of the early stage of colorectal carcinogenesis.^31^ ^32^ Although there were no significant differences in the total number of ACF and ACs between the WT and KO littermates mice under the basal diet (data not shown), the numbers of ACF and ACs in the KO mice were significantly higher than those in the WT littermates under the high-fat diet (fig 2A,B). The macroscopic and microscopic characteristics of the ACF in the WT and KO mice under the high-fat diet condition are shown in fig 2C,D; no morphological differences of the ACF were observed between the WT and the KO mice. The differences in the body weight (supplementary fig 4C,D) and the serum levels of adiponectin, glucose, insulin, lipids, and tumour necrosis factor α between the WT and the KO mice are shown in supplementary table 2. In agreement with previous metabolic studies of adiponectin-deficient mice,^33^ there were no differences between the two groups under the high-fat diet. To confirm the protective role of adiponectin in colorectal carcinogenesis, the food-borne carcinogen PhIP was used as a second model of colon carcinogenesis in mice fed a high-fat diet. Similar results to those obtained using the AOM-induced carcinogenesis model was obtained (supplementary fig 2).

### Increase in cell proliferative activity in adiponectin-deficient mice under the high-fat diet condition

We investigated the proliferative activity of the colon epithelium by determining the BrdU and PCNA labelling indices. Both indices were increased in the KO mice as compared with their WT littermates under the high-fat diet (fig 3A–D). On the other hand, there was no difference under the basal diet (data not shown). Moreover, we examined both indices in colon polyps under the high-fat diet but there was no difference between WT and KO mice (supplementary fig 5).

### Adiponectin ameliorates epithelial cell hyper-proliferation in adiponectin-deficient mice under the high-fat diet condition

We administered recombinant adiponectin via an intraperitoneal injection to KO mice in comparison to their WT littermates under the high-fat diet condition. Globular domain adiponectin exerted a more potent effect on the suppression of proliferative activity of colon epithelial cells than full-length adiponectin under the high-fat diet (fig 3E). The same effect was observed on the suppression of ACF formation (fig 3F).

### The increase in cell proliferative activity in adiponectin receptor 1-deficient mice under the high-fat diet

By using western blot analyses we investigated whether adiponectin receptors are expressed in the colon epithelium, and observed that AdipoR1 was predominantly expressed in the colon in comparison to AdipoR2 (fig 4A).

The BrdU index in the *AdipoR1−/−* mice was significantly higher than in their WT littermates under the high-fat diet (fig 4B). The numbers of ACF and ACs in the *AdipoR1−/−* mice were also significantly higher than their WT littermates (fig 4D). However, no difference in BrdU index and the number of ACF was observed in *AdipoR2−/−* mice and their WT littermates (fig 4C,E). These results suggest that the AdipoR1-mediated, but not the AdipoR2-mediated, pathway may play an important role in the suppressive effect of adiponectin on colorectal carcinogenesis under the high-fat diet, not under the basal diet.

### The mTOR pathway is relatively activated in the colon epithelium of adiponectin-deficient mice in comparison to wild-type mice under the high-fat diet

**Figure 5:**
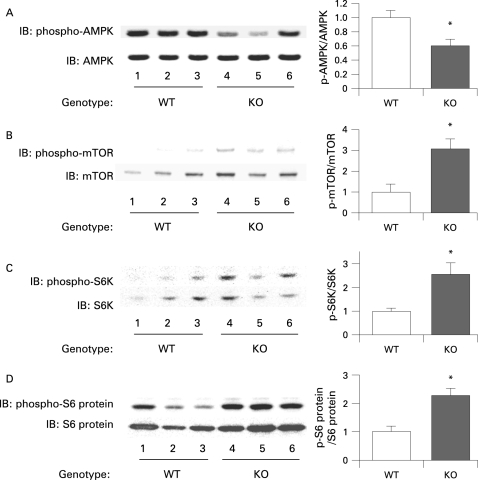
Activation of the mammalian target of rapamycin (mTOR) pathway in adiponectin-deficient (KO) mice compared with that in wild-type (WT) mice under the high-fat diet condition. Western blot analysis for phosphorylated and total AMP-activated protein kinase (AMPK) (A), mTOR (B), p70 S6 kinase (C) and S6 protein (D) in the colon from the WT and KO mice under the high-fat diet condition. Left panels: Pictures of the western blotting. Right panels: Graphs showing the ratios of the phosphorylated protein to the total protein. Each column represents the mean (with the SEM), *p<0.05.

**Figure 6:**
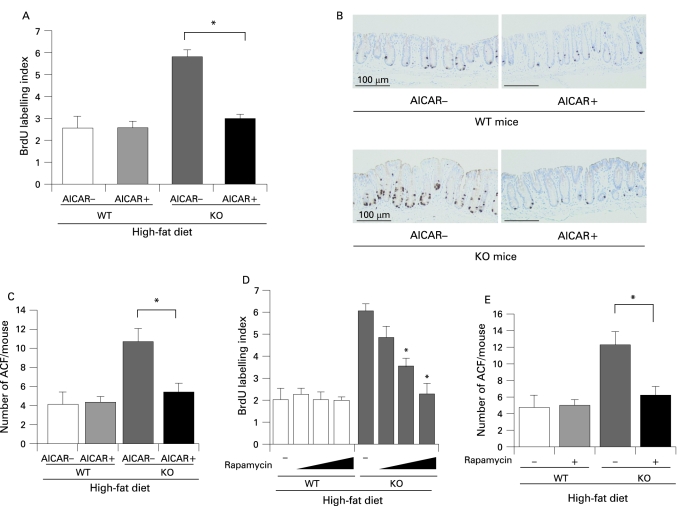
Suppression of epithelial cell hyper-proliferation by activation of AMP-activated protein kinase (AMPK) or by inhibition of the mammalian target of rapamycin (mTOR) in adiponectin-deficient (KO) mice under the high-fat diet condition. Wild-type (WT) mice (6 weeks old) were injected intraperitoneally with the AMPK activator 5-aminoimidazole-4-carboxamide-l-β-d-ribofuranoside (AICAR) (0.1 mg/kg/day), or vehicle until the end of the experiment. Mice in the high-fat diet group were also given two weekly intraperitoneal injections of 10 mg/kg of azoxymethane (AOM). (A) Average bromodeoxyuridine (BrdU) labelling index in the WT and KO mice treated (+) or not treated (−) with AICAR under the high fat diet condition. BrdU was administered intraperitoneally 1 h prior to the sacrifice of the animals. Each column represents the mean (with the SEM), *p<0.05. (B) Representative immunohistochemical staining patterns for BrdU in each group. (C) Average number of aberrant crypt foci (ACF) in the WT and KO mice treated (+) or not treated (−) with AICAR under the high fat diet condition. (D) WT and KO mice fed the high-fat (HF) diet were injected intraperitoneally with various doses of rapamycin (0.2, 0.4, 0.8 mg/kg) or only with vehicle every other day for 6 weeks. Average values of the BrdU index were decreased in the KO mice in a dose dependent manner, but not in the WT mice. *p<0.05 compared to non-treated KO mice. (E) Average number of ACF in the WT and KO mice treated (+) or not treated (−) with rapamycin (0.8 mg/kg) under the high fat diet condition. Each bar represents the mean (with the SEM), *p<0.05.

In order to clarify the mechanisms underlying the enhanced proliferative activity of the colon epithelial cells in the presence of adiponectin deficiency, we investigated the expression levels of various potential target proteins in colonic specimens prepared from the WT mice and KO mice under the high-fat diet. The results of western blot analysis revealed that the amounts of phosphorylated mTOR, S6 kinase and S6 protein were significantly higher in the KO mice compared with the WT mice under the high-fat diet (fig 5B–D). It has been reported that adiponectin activates AMPK via AdipoR1, and AMPK is known to suppress the mTOR pathway.^20^ ^34^ A significant decrease in the level of phosphorylated AMPK was observed in the KO mice compared with that in the WT mice under the high-fat diet (fig 5A). Moreover, adiponectin administration ameliorated activation of the AMPK/mTOR pathway in KO mice under the high-fat diet condition (supplementary fig 6). These results indicate that, under the high-fat diet, deficiency of adiponectin suppresses AMPK activation, which results in activation of the mTOR pathway directly involved in cell proliferation. To confirm whether adiponectin actually suppresses the AMPK/mTOR pathway, we treated mice with the specific AMPK activator AICAR, or the mTOR inhibitor rapamycin. The increase of cell proliferation in the colon epithelium was significantly suppressed by AICAR in the KO mice under the high-fat diet, but no effect in WT mice under high-fat diet (fig 6A,B). Similarly, ACF formation was significantly suppressed by AICAR in the KO mice under the high-fat diet, but not in the WT mice (fig 6C). In the KO mice under the high-fat diet, treatment with rapamycin significantly reduced the BrdU index in a dose dependent manner and ACF formation (fig 6D,E). These results indicate that the activation of the mTOR pathway may play important roles in the increase in epithelial cell proliferation in KO mice under the high-fat diet, and may play an important role in the promotion of colon carcinogenesis in KO mice under the high-fat diet condition.

## DISCUSSION

The existence of a relationship between high-fat diets and colorectal cancer has been speculated for a long time, but no definitive conclusions have been arrived at yet.^35^ It has been reported that the secretion of adiponectin from adipocytes is suppressed in obese humans.^11^ Considered together with the knowledge that obesity is also an important risk factor for colorectal cancer,^4^ we speculated that adiponectin might suppress the development of colorectal cancer.

We demonstrated significantly enhanced formation of polyps and ACF in the KO mice compared with that in the WT mice under the high-fat diet. Furthermore, an increase in proliferative activity of colonic epithelial cells was also observed in the KO mice under the high-fat diet, but not under the basal diet. These results suggest that under the high-fat diet, but not under basal diet, a deficiency of adiponectin significantly promotes the proliferative activity of the colonic epithelial cells, and thereby may be promoting colorectal carcinogenesis. We demonstrated that the AdipoR1 is predominantly expressed in colon epithelium. The increase in the proliferative activity of the colonic epithelial cells and the number of ACF were observed in AdipoR1*−*/*−* mice, not in the AdipoR2*−*/*−* mice, under high-fat diet condition. These results suggest that the AdipoR1-mediated, but not the AdipoR2-mediated, pathway may play an important role in the suppressive effect of adiponectin on the increased in epithelial cell proliferation under the high-fat diet.

We demonstrated the activation of the mTOR pathway and inactivation of AMPK in colon epithelial cells in the KO mice under the high-fat diet, but there was no difference under the basal diet (data not shown). Moreover, the replacement of adiponectin ameliorated the activated mTOR pathway by adiponectin deficiency. AICAR, the AMPK specific activator, suppressed the increase in epithelial cell proliferation in KO mice, but not in WT littermates, under high-fat diet. Furthermore, rapamycin, an mTOR inhibitor, also significantly suppressed the increase in epithelial cell proliferation only in KO mice under high-fat diet in a dose dependent manner, suggesting that mTOR plays an important role in promoting epithelial cell proliferation where there is a lack of adiponectin under a high-fat diet. It has been reported that AMPK directly inhibits mTOR.^28^ Therefore we speculate that the AMPK/mTOR pathway is a possible mechanism closely involved in the protective effect of adiponectin in colon carcinogenesis under the high-fat diet (supplementary fig 7). Concerning other major pathways in the carcinogenesis, it was reported that adiponectin attenuated the adenomatous polyposis coli (APC)/β-catenin pathway,^36^ and increased p53 expression^37^ in different kinds of cancer cells. p53 also suppresses the mTOR pathway through activation of phosphatase and tensin homologue deleted on chromosome ten (PTEN), AMPK, insulin-like growth factor-1-binding protein 3 (IGF1-BP3) and tuberous sclerosis complex-2 (TSC-2).^38^ Although the mechanism underlying the promotion of colon carcinogenesis by a high-fat diet is still unknown, our present data strongly suggest that plasma adiponectin derived from adipocytes suppresses the mTOR pathway through the activation of AMPK, resulting in suppression of the cell proliferative activity and, thereby, suppression of colon carcinogenesis, under the high-fat diet. However, in the event of a decrease in plasma adiponectin level, AMPK activity is suppressed, resulting in the activation of mTOR and the members downstream in the pathway, such as the p70 S6 kinase and S6 protein. We speculate that activation of the mTOR pathway directly promotes colonic epithelial cell proliferation and, thereby, colorectal carcinogenesis.

It has been reported that the plasma adiponectin levels are decreased in humans under the conditions of obesity and/or diabetes mellitus.^11^ However, it was reported that plasma levels of adiponectin in the mice are not decreased in response to high-fat feeding for several weeks.^39^ Therefore, we used adiponectin-deficient mice to elucidate the role of adiponectin on colonic epithelial proliferation and carcinogenesis under the high-fat diet. Our experimental condition in which we used adiponectin-deficient mice fed a high-fat diet may well have reflected these pathophysiological conditions in humans.

The purpose of our study was to elucidate the role of adiponectin on colon carcinogenesis, not to elucidate the mechanism whereby a high-fat diet promotes carcinogenesis. This mechanism remains unknown. However, we could provide a possible mechanism underlying the protective roles of adiponectin in colorectal carcinogenesis promoted by a high-fat diet We consider that AMPK and mTOR may be novel therapeutic targets for the prevention of colorectal cancer under the low levels of plasma adiponectin in an obese population where the obesity is a result of a Western-style diet with a high fat content. Our results shed light on a novel mechanism by which adiponectin might suppress carcinogenesis mediated by a high-fat diet. Continued investigation to elucidate the precise mechanisms involved is necessary because of the major clinical implications.
